# TIM-3 expression in tumor and stromal cells are associated with the prognosis in patients with epithelial ovarian cancer

**DOI:** 10.1097/MD.0000000000044921

**Published:** 2025-10-10

**Authors:** Jianlei Wu, Yaru Zhang, Haibo Zhang, Yan Li, Zhihui Jie, Shan Kang

**Affiliations:** a Department of Gynecological Oncology, Shandong Cancer Hospital and Institute, Shandong First Medical University and Shandong Academy of Medical Sciences, Jinan, China; b Department of Obstetrics and Gynaecology, Hebei Medical University, Fourth Hospital, Shijiazhuang, China; c Department of Obstetrics and Gynaecology, The Second Affiliated Hospital of Shandong First Medical University, Tai’an, China.

**Keywords:** epithelial ovarian cancer, prognosis, TIM-3, tumor cells, tumor microenvironment

## Abstract

T-cell immunoglobulin and mucin domain-containing molecule 3 (TIM-3) has been reported to be overexpressed and associated with poor prognosis in solid tumors. However, its features and prognostic value in epithelial ovarian cancer (EOC) remain undetermined. In this study, we aimed to characterize TIM-3 expression and its prognostic significance in patients with EOC. A total of 134 EOC patients and 20 healthy controls from North China were included. TIM-3 mRNA and protein expression in EOC tumor tissues and benign ovarian tissues were detected by real-time quantitative PCR and immunohistochemistry. The distribution of TIM-3 protein in different regions of EOC tissue (tumor cells and the tumor microenvironment) were evaluated by multicolor immunofluorescence. Associations between their expression and clinicopathological parameters as well as survival analyses were performed. The results showed that high expression levels of TIM-3 mRNA were significantly associated with shorter progression-free survival (PFS; *P* < .001, hazard ratio [HR] = 1.57, 95% confidence interval [CI] = 1.29–1.91) and overall survival (OS; *P* = .013, HR = 1.31, 95% CI = 1.06–1.63) durations in EOC patients. High TIM-3 expression levels in tumor cells had shorter PFS (HR = 1.62, 95% CI = 1.09–2.46, *P* = .018) and OS (HR = 1.81, 95% CI = 1.19–2.75, *P* = .006) compared with those low TIM-3 expression levels. Similarly, TIM-3 in the tumor microenvironment was also an independent factor that affected the clinical outcome of EOC patients (PFS: HR = 1.99, 95% CI = 1.29–3.08, *P* = .002; OS: HR = 2.13, 95% CI = 1.37–.30, *P* = .001). These findings indicated that IM-3 may be a potential biomarker for predicting prognosis and immunotherapy efficacy in patients with EOC, exerting different roles on tumor cells and tumor microenvironment.

## 1. Introduction

Epithelial ovarian cancer (EOC) is the most common cause of cancer-related death associated with gynecological cancer.^[1]^ Due to the lack of specific tumor markers and early symptoms, >70% of EOC patients are diagnosed at an advanced stage.^[2]^ At present, the standard of care for EOC is based on surgery and platinum-based chemotherapy. Although adjuvant therapy strategies such as PARP inhibitors have also been used to treat EOC, the 5-year survival rate remains approximately 40% for advanced EOC, with little improvement.^[3]^ In recent years, as the examination of the tumor microenvironment has become more comprehensive, an increasing number of clinical trials focusing on immunotherapy for EOC have emerged, such as trials of antibodies against PD-1/PD-L1.^[4]^ However, despite these advancements, immunotherapy continues to fall short of delivering a satisfactory response rate.^[5,6]^ Hence, it is imperative to comprehend the tumor microenvironment to identify novel potential markers that can accurately predict the outcomes of immunotherapy. Furthermore, the discovery of specific molecular signatures that can be effectively targeted by immunotherapy is crucial for enhancing the prognosis of EOC patients.

T-cell immunoglobulin mucin domain molecule 3 (TIM-3), an immune checkpoint molecule, has been shown to be expressed on dendritic cells, natural killer cells (NK cells), CD4/8 + T lymphocytes and several kinds of tumor cells.^[7–10]^ Evidences indicated that TIM-3 may have a direct role in promoting tumorigenesis, proliferation and invasion, as has been observed in tumor cells, or driving immunosuppression.^[11–14]^ Previous studies of patients with EOC have found that high expression levels of TIM-3 on tumor-infiltrating Tregs is associated with larger tumor size and poor prognosis.^[15]^ Further studies have reported that the expression level of TIM-3 in peripheral blood CD4 + and CD8 + T cells in patients with EOC is elevated compared with that in healthy controls, and the clinical stage and tumor grade of patients with high expression levels of TIM-3 are significantly higher than those with low expression levels.^[16]^ Our previous study also showed that EOC patients with the rs10053538 CA + AA genotype of the TIM-3 gene had worse progression-free survival (PFS) and overall survival (OS) than those with the CC genotype.^[17]^ These data suggested that TIM-3 might play an important role in the occurrence and progression of EOC. However, the expression of TIM-3 in EOC tissues and its relationship with the prognosis of EOC patients have not yet been clarified. Therefore, it is important to determine the distribution and function of TIM-3 in EOC for the application of anti-TIM-3 immunotherapy in patients with EOC.

Currently, TIM-3 blockade therapy is considered to have high potential in combination with anti-PD-1/L1 therapy and may overcome resistance to anti-PD-1/L1 therapy. Even more importantly, several anti-TIM-3 monoclonal antibodies are currently in clinical trials.^[18,19]^ In the following study, we initially assessed TIM-3 mRNA and protein expression in EOC tumor tissues and benign ovarian tissues by real-time quantitative PCR (RT-qPCR) and immunohistochemistry (IHC). We further evaluated the distribution of TIM-3 protein in different regions of EOC tissue (tumor cells and the tumor microenvironment) by multicolor immunofluorescence and examined their correlation with prognosis in patients with EOC.

## 2. Materials and methods

### 2.1. Patients and tissue specimens

A total of 134 EOC tumor samples were collected from patients who underwent surgery in the Fourth Hospital of Hebei Medical University from January 2009 to January 2015. Inclusion criteria were as follows: pathologically confirmed EOC and standard chemotherapy treatment after surgery. Exclusion criteria were as follows: patients with recurrence, patients who had received other treatments before surgery (neoadjuvant chemotherapy, endocrine therapy, radiotherapy, etc), patients with a history of tumors in other sites, patients with immune system diseases, and patients with systemic infection. Standard postchemotherapy surveillance included serial physical examinations, serum CA-125 level testing, and computed tomography scanning as clinically indicated. According to the NCCN guidelines, recurrent disease was identified by clinical symptoms (i.e., pelvic pain and weight loss), blood biomarkers (i.e., elevated CA-125 levels), and/or imaging examinations.^[20]^ For each case, the diagnosis was confirmed by 2 senior pathologists through a review of HE-stained slides. During the same period, normal ovarian tissues (n = 20) were collected from patients who underwent hysterectomy for benign uterine disease. All EOC patients who received platinum-based chemotherapy after primary cytoreductive surgery were followed up from August 2009 to April 2022. PFS was defined as the time from the date of surgery to the 1st recurrence or last follow-up. OS was defined as the time from diagnosis to all-cause death. The study was approved by the Ethics Committee of the Fourth Hospital of Hebei Medical University (No. 2015MEC005), and informed consent was obtained from all of the recruited subjects according to the Helsinki Declaration.

### 2.2. Real-time quantitative PCR

Total RNA extraction and cDNA synthesis from 120 ovarian cancer tissues were performed as previously described.^[17]^ RT-qPCR was carried out using SYBR-Green II Premix (Takara, Dalian, China) with the ABI 7500 detection system (Applied Biosystems, Carlsbad). The reaction conditions were as follows: 95°C for 30 seconds and 40 cycles of 95°C for 5 seconds, 60°C for 30 seconds, and 72°C for 5 seconds. The primers were synthesized by Sangon Biotech Co., Ltd. (Shanghai, China), and the primer sequences were as follows: TIM-3, forward 5′-CTACATG GAGCAGGGAT-3′ and reverse 5′-CTGAGGGAGGGAGGTTG-3′, and GAPDH, forward 5′-ACCACGTCCATGCCATCAC-3′ and reverse 5′-TCCACCACCCTGTTGCTG TA-3′. GAPDH was used as an internal control. The relative TIM-3 mRNA level was calculated by the 2^−ΔΔCt^ method, and each reaction was performed in triplicate.

### 2.3. Gene expression analysis of TIM-3 in the GEPIA database

We analyzed TIM-3 mRNA levels in ovarian cancer tissues and normal ovarian tissues using the GEPIA database (http://gepia.cancer-pku.cn/index.html), which contains data on approximately 9736 tumors and 8587 normal samples from the The Cancer Genome Atlas database and GTEx RNA-sequencing projects. In this study, Student *t* test was used to generate a *P* value for the TIM-3 expression profiles through GEPIA.^[21]^ The cutoff value of log_2_FC was set as 1, and the *P* value was set to .01.

### 2.4. Immunohistochemistry

IHC staining of paraffin-embedded tissue sections was processed according to a standard protocol. Anti-TIM-3 antibody (ab241332, 1:1000) was purchased from Abcam (Cambridge, UK). The results of IHC staining were scored and judged by experienced pathologists in a double-blind situation with an Olympus BX-51 microscope (Olympus, Waltham). If there was uncertainty, a 3rd pathologist made the judgment. In this study, the TIM-3 positivity and staining score were defined according to a previous study.^[18]^ Five high-power fields were randomly selected for observation in each section. The established staining score (range, 0–7) was the sum of staining intensity and proportion of positively stained cells. A final score of 0 to 4 points indicated no or low expression levels, and 5 to 7 points indicated high expression levels.

### 2.5. Multicolor immunofluorescence staining (mIF) and interpretation

After whole-slide examination by a trained gynecological oncopathologist, three 0.6-mm diameter cores were collected from tissue blocks for inclusion in a tissue microarray.

The operation steps for mIF were the same as those for IHC, except that each antigen retrieval step was performed with antigen retrieval buffer, which can remove the previous primary and secondary antibodies. After cooling and washing, the slides were incubated with the following primary antibodies: anti-TIM-3 antibody (ab241332, 1:1000, Abcam, Cambridge, UK)^[22]^ and anti-panCK (PA125, Baidao Medical Technology, Suzhou, China). The slides were counterstained with DAPI, and then images and data were processed by TissueGnostics image processing software. The following observation results and image scanning results were used to determine the positive judgment value (cutoff value) of each antigen staining experiment and verify the composite of each single-color fluorescence experiment to ensure that there was no spectral overlap between each single-color fluorescence experiment. The TIM-3-positive cells were counted in different regions (tumor cells and the tumor microenvironment) of EOC tissue based on CK expression (CK+ region and CK− region). mIF and image processing were completed by Shanghai Biochip Co., Ltd., Shanghai, China and Shanghai National Engineering Research Center for Biochips.

### 2.6. Statistical analysis

Statistical analyses were carried out using SPSS 24.0 (SPSS Inc., Chicago) and GraphPad Prism 8.0 software (San Diego, http://www.graphpad.com). Continuous variables were expressed as mean ± standard deviation. Differences between 2 groups were compared by a *t* test or the Wilcoxon rank sum test. Categorical variables were expressed as relative distribution frequencies (percentages), and differences between 2 groups were compared by the χ2 test or Fisher exact probability method. The R3.5.0 software (R Core Team, 2018) survival receiver operating characteristic (ROC) package was used to analyze the time-ROC selected cutoff values, the survival and survminer packages were used for survival analysis, the Kaplan–Meier method was used to draw the survival curves, and the log-rank χ2 test was used to compare survival rates between groups. The Cox proportional hazard regression model was used to obtain the adjusted hazard ratio (HR) and 95% confidence interval (CI). The difference was statistically significant at *P* < .05.

## 3. Results

### 3.1. Baseline information

The median age of the patients was 56 years (range 21–77), and the median age of the control group was 45 years (range 40–81). According to FIGO staging, 9 (6.72%) cases were stage I, 28 (20.90%) cases were stage II, 84 (62.69%) cases were stage III, and 13 (9.70%) cases were stage IV. According to the histological diagnostic criteria proposed by the WHO, there were 91 (67.91%) cases of serous carcinoma, 32 (23.88%) cases of endometrioid carcinoma, 5 (3.73%) cases of mucinous carcinoma, 4 (2.99%) cases of clear cell carcinoma, and 2 (1.49%) cases of mixed carcinoma. According to the pathological grading standard, 27 (20.15%) cases were grade G1, 36 (26.87%) cases were grade G2, and 71 (52.99%) cases were grade G3.

### 3.2. High TIM-3 expression levels were associated with poor prognosis in patients with EOC

**Figure 1.**
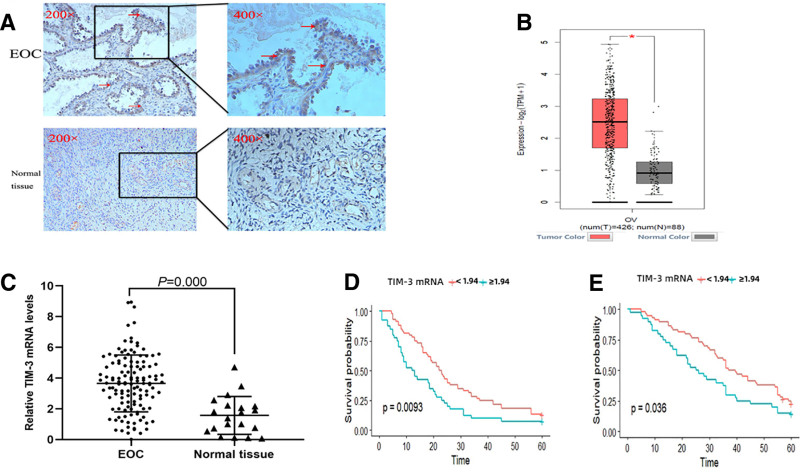
TIM-3 expression in ovarian tumor and normal tissue and relationship between TIM-3 mRNA expression and prognosis in EOC patients. (A) Expression of TIM-3 in EOC and normal ovarian tissues. (B) The differential expression of TIM-3 in ovarian tumor and normal ovarian tissues was analyzed by GEPIA. Red color means ovarian cancer tissues and gray color means normal tissues. (C) The mRNA expression of TIM-3 in EOC samples (Tumor) and normal ovarian tissues (Normal) was tested by qRT-PCR. (D, E) Kaplan–Meier estimates of progression-free survival (PFS) in patients categorized by TIM-3 mRNA expression. EOC = epithelial ovarian cancer, TIM-3 = T-cell immunoglobulin and mucin-domain containing-3.

To assess TIM-3 protein expression, we examined 46 EOC tissues and 20 normal ovarian tissues by IHC. The results showed that TIM-3 protein was localized to the cell membrane and cytoplasm, with a brownish yellow granular distribution (Fig. 1A). The positive expression rate of TIM-3 was 76.1% (35/46) in the EOC group and 25.0% (5/20) in the normal ovarian tissue group. The frequency of positive TIM-3 expression in EOC was significantly higher than that in normal ovarian tissue (χ2 = 13.173, *P* < .001).

We found that TIM-3 mRNA expression levels in EOC samples were approximately 1.4 times those in normal ovarian tissues by the GEPIA website based on the The Cancer Genome Atlas and GTEx databases (Fig. 1B). RT-qPCR results also showed that the expression levels of TIM-3 mRNA in EOC tissues were significantly higher than those in normal ovarian tissues (*t* = 4.826, *P* = .000; Fig. 1C). Moreover, Kaplan–Meier analysis was used to evaluate the relationship between TIM-3 mRNA expression and the prognosis of patients with EOC. The results showed that the median PFS results for patients with high and low expression levels were 12 and 19.3 months, respectively, and the mean OS values of those patients were 38.9 and 47.8 months, respectively. Kaplan–Meier plots illustrated the differences in PFS (Fig. 1D; *P* = .0093) and OS (Fig. 1E; *P* = .036) for EOC patients categorized by different expression levels of TIM-3 mRNA.

### 3.3. Relationship between TIM-3 protein expression and clinicopathological parameters in different regions

**Figure 2.**
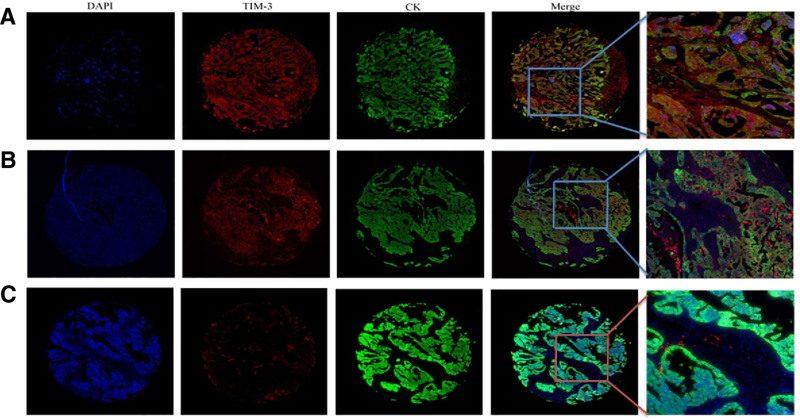
mIF staining analysis of TIM-3 and CK in ovarian cancer tissues. (A) The protein expression of TIM-3 in CK+ region. (B) The protein expression of TIM-3 in CK− region. (C) TIM-3 protein was expressed negatively. Single staining libraries representing DAPI, TIM-3, and CK were constructed respectively, and then a composite image was created and viewed in a localized area. mIF = multicolor immunofluorescence staining, TIM-3 = T-cell immunoglobulin and mucin-domain containing-3.

In this study, CK labeling was used to distinguish tumor cells (CK+) from the tumor microenvironment (CK−) in EOC. The R3.5.0 software survival ROC package was used to analyze the expression of TIM-3 in EOC tissue to select the cutoff value. After analysis, for the expression of TIM-3 protein in tumor cells, the cutoff value was 8.14% (the percentage of TIM-3-positive cells in EOC tissue was 8.14%), and for the expression of TIM-3 protein in the tumor microenvironment, the cutoff value was 0.98% (the percentage of TIM-3-positive cells in EOC tissue was 0.98%). Table 1 shows patients’ clinical characteristics stratified by the level of TIM-3 expression in tumor cells and the tumor microenvironments. Figure 2 shows the results of mIF staining analysis of TIM-3 and CK in the EOC tissues: the expression of TIM-3 protein in the CK+ region (Fig. 2A); the expression of TIM-3 protein in the CK− region (Fig. 2B); and negative expression of TIM-3 protein (Fig. 2C).

**Table 1:** Association between TIM-3 expression and clinical characteristics of EOC patients.

Group	TIM-3 in CK+ region		χ2	*P*	TIM-3 in CK− region		χ2	*P*
	Low	High			Low	High		
Age								
< 50	26	29	0.057	.811	23	32	0.185	.667
≥ 50	39	40			36	43		
Stage								
I–II	26	11	9.692	.002	26	11	14.281	.000
III–IV	39	58			33	64		
Grade								
1–2	37	26	4.975	.026	34	29	4.766	.029
3	28	43			25	46		
Clinical histology								
Serous	44	47	2.755	.252	40	51	0.368	.832
Endometrioid	18	14			15	17		
Others	3	8			4	7		
Residual tumor								
0 cm	19	19	11.331	.003	17	21	5.551	.062
≤ 1cm	38	25			33	30		
> 1 cm	8	25			9	24		

EOC = epithelial ovarian cancer, TIM-3 = T-cell immunoglobulin and mucin-domain containing-3.

### 3.4. TIM-3 expression in different regions is associated with prognosis in patients with EOC

**Figure 3.**
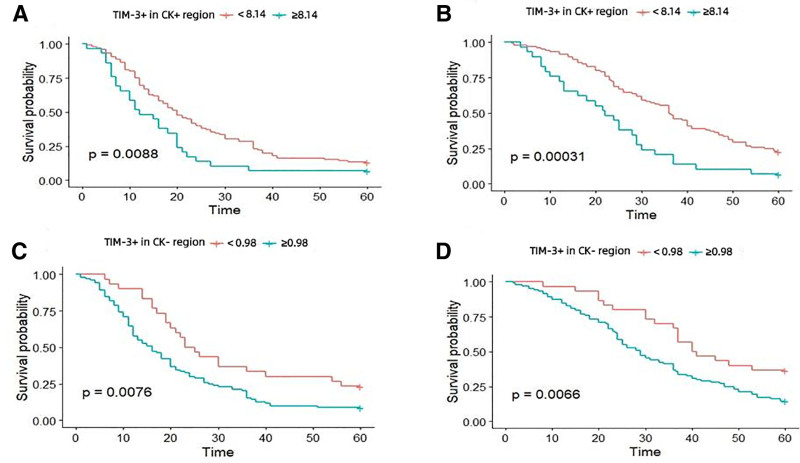
Relationship between TIM-3 mRNA expression and prognosis in EOC patients. (A, B) The protein expression of TIM-3 in CK+ region influencing the PFS and OS. (C, D) The protein expression of TIM-3 in CK− region influencing the PFS and OS. EOC = epithelial ovarian cancer, OS = overall survival, PFS = progression-free survival, TIM-3 = T-cell immunoglobulin and mucin-domain containing-3.

The 5-year survival rate of 134 patients with EOC was 21.6%, and the recurrence rate was 88.1%. The impact of TIM-3 expression in tumor cells and the tumor microenvironment on the prognosis of patients with EOC was investigated using the survival and survminer packages (R3.5.0). As presented in Figure 3A–B, the median PFS values of EOC patients with high and low expression levels of TIM-3 in tumor cells were 19 and 13 months, respectively, and the mean OS values of these patients were 44 and 27 months. Kaplan–Meier plots illustrated that compared with patients with low expression levels, patients with high TIM-3 expression levels in tumor cells had shorter PFS (Fig. 3A, *P* = .0088) and OS (Fig. 3B, *P* = .00031). After adjusting for prognostic factors (age, FIGO stage, grade, tumor residual size and histology), patients with high TIM-3 expression levels had an increased risk of disease progression (HR = 1.62, 95% CI = 1.09–2.46, *P* = .018) and death (HR = 1.81, 95% CI = 1.19–2.75, *P* = .006) compared with those with low TIM-3 expression levels (Table 2).

**Table 2:** Multivariate analysis of the relationship between TIM-3 protein expression in CK+ region and prognosis in EOC patients.

Group	PFS				OS			
	Univariate model		Multivariate model		Univariate model		Multivariate model	
	HR (95% CI)	*P*	HR (95% CI)	*P*	HR (95% CI)	*P*	HR (95% CI)	*P*
Age								
< 50	reference		reference		reference		reference	
≥ 50	1.31 (0.91–1.90)	.145	1.05 (0.71–1.55)	.814	1.19 (0.81–1.75)	.368	0.86 (0.57–1.29)	.471
Stage								
I–II	reference		reference		reference		reference	
III–IV	2.49 (1.60–3.88)	<.001	2.08 (1.28–3.38)	.003	2.99 (1.83–4.89)	<.001	2.77 (1.62–4.75)	<.001
Grade								
1–2	reference		reference		reference		reference	
3	1.23 (0.85–1.76)	.270	1.52 (1.03–2.25)	.036	1.19 (0.81–1.74)	.370	1.40 (0.94–2.09)	.099
Clinical histology								
Serous	reference		reference		reference		reference	
Endometrioid	0.43 (0.27–0.68)	<.001	0.41 (0.25–0.66)	<.001	0.49 (0.31–0.80)	.004	0.50 (0.30–0.81)	.005
Others	0.52 (0.26–1.04)	.064	0.49 (0.24–1.00)	.050	0.54 (0.25–1.16)	.114	0.50 (0.23–1.11)	.089
Residual tumor								
0 cm	reference		reference		reference		reference	
≤ 1 cm	0.93 (0.55–1.57)	.785	1.15 (0.67–1.99)	.605	0.98 (0.55–1.74)	.947	1.39 (0.77–2.51)	.279
>1 cm	2.17 (1.35–3.46)	.001	2.43 (1.48–4.01)	<.001	2.73 (1.65–4.51)	<.001	3.29 (1.94–5.60)	<.001
TIM-3 in CK+ region								
Low	reference		reference		reference		reference	
High	1.57 (1.09–2.26)	.016	1.64 (1.09–2.46)	.018	1.69 (1.15–2.49)	.007	1.81 (1.19–2.75)	.006

CI = confidence interval, EOC = epithelial ovarian cancer, HR = hazard ratio, OS = overall survival, PFS = progression-free survival, TIM-3 = T-cell immunoglobulin and mucin-domain containing-3.

We further investigated the relationship between the expression of TIM-3 in the tumor microenvironment and the prognosis of EOC patients. The results showed that the median PFS and OS of EOC patients with high TIM-3 expression levels in the tumor microenvironment were 13 and 25 months, respectively. However, the median PFS and OS of EOC patients with low TIM-3 expression levels were 22 and 41 months, respectively. Kaplan–Meier plots illustrated that patients with high TIM-3 expression levels in the tumor microenvironment had shorter PFS (Fig. 3C, *P* = .0076) and OS (Fig. 3D, *P* = .0066) than patients with low TIM-3 expression levels. Multivariate analyses for each potential predictor of survival using Cox regression showed that TIM-3 in the tumor microenvironment was an independent factor that affected the clinical outcome of EOC patients (PFS: HR = 1.99, 95% CI = 1.29–3.08, *P* = .002; OS: HR = 2.13, 95% CI = 1.37–3.30, *P* = .001; Table 3). These findings suggested that high TIM-3 expression levels in the tumor microenvironment was associated with poor prognosis in EOC patients. Therefore, it can be assumed that TIM-3 plays a key role in the development of EOC tumors through the interaction between the tumor microenvironment and tumor stroma.

**Table 3:** Multivariate analysis of the relationship between TIM-3 protein expression in CK− region and prognosis in EOC patients.

Group	PFS				OS			
	Univariate model		Multivariate model		Univariate model		Multivariate model	
	HR (95% CI)	*P*	HR (95% CI)	*P*	HR (95% CI)	*P*	HR (95% CI)	*P*
Age								
< 50	reference		reference		reference		reference	
≥ 50	1.31 (0.91–1.90)	.145	1.13 (0.76–1.67)	.541	1.19 (0.81–1.75)	.368	0.93 (0.62–1.40)	.728
Stage								
I–II	reference		reference		reference		reference	
III–IV	2.49 (1.60–3.88)	<.001	2.04 (1.26–3.29)	.004	2.99 (1.83–4.89)	<.001	2.76 (1.62–4.71)	<.001
Grade								
1–2	reference		reference		reference		reference	
3	1.23 (0.85–1.76)	.270	1.54 (1.04–2.26)	.029	1.19 (0.81–1.74)	.370	1.40 (0.94–2.08)	.095
Clinical histology								
Serous	reference		reference		reference		reference	
Endometrioid	0.43 (0.27–0.68)	<.001	0.40 (0.25–0.64)	<.001	0.49 (0.31–0.80)	.004	0.50 (0.31–0.82)	.005
Others	0.52 (0.26–1.04)	.064	0.39 (0.19–0.82)	.012	0.54 (0.25–1.16)	.114	0.41 (0.18–0.93)	.032
Residual tumor								
0 cm	reference		reference		reference		reference	
≤ 1cm	0.93 (0.55–1.57)	.785	1.31 (0.75–2.29)	.341	0.98 (0.55–1.74)	.947	1.56 (0.86–2.90)	.140
>1 cm	2.17 (1.35–3.46)	.001	3.12 (1.80–5.39)	<.001	2.73 (1.65–4.51)	<.001	4.14 (2.34–7.33)	<.001
TIM-3 in CK− region								
Low	reference		reference		reference		reference	
High	1.57 (1.09–2.26)	.041	1.99 (1.29–3.08)	.002	1.69 (1.15–2.49)	.007	2.13 (1.37–3.30)	.001

CI = confidence interval, EOC = epithelial ovarian cancer, HR = hazard ratio, OS = overall survival, PFS = progression-free survival, TIM-3 = T-cell immunoglobulin and mucin-domain containing-3.

## 4. Discussion

This study investigated the association between TIM-3 expression status in EOC tumor tissue and clinical outcomes. The results showed that high expression levels of TIM-3 mRNA were significantly associated with shorter PFS and OS durations in EOC patients. To further verify this result, we analyzed the relationship between the expression level of TIM-3 protein in different areas of tumor tissue and the clinical prognosis in patients with EOC by multicolor immunofluorescence. The results also showed that patients with high expression levels of TIM-3 had worse PFS and OS durations than those with low expression levels, regardless of TIM-3 expression in tumor cells or the tumor microenvironment. To our knowledge, this is the 1st study to evaluate the expression status of TIM-3 in EOC tumor cells and the tumor microenvironment and its role in the clinical prognosis of patients with EOC.

Accumulated evidence has shown that the expression of TIM-3 is upregulated in several kinds of tumor cells, including cervical cancer,^[23]^ gastric cancer,^[24]^ and renal cell carcinoma.^[25]^ Recently, a meta-analysis showed that high expression levels of TIM-3 in tumor cells is closely related to poor PFS and OS in patients with digestive system cancer and respiratory system cancer.^[10]^ These studies strongly revealed the potential role of TIM-3 in tumor progression. In our previous study, we used Kaplan–Meier plotter database analysis to show that overexpression of TIM-3 mRNA is associated with poor clinical outcomes in patients with EOC.^[17]^ In this study, we confirmed the high expression levels of TIM-3 in EOC tissues by IHC and RT-qPCR and again determined that high expression of TIM-3 mRNA was related to shorter PFS and OS in EOC patients. Furthermore, we detected TIM-3 protein expression in tumor cells (CK+) and the tumor microenvironment (CK−) by multiple immunofluorescence and analyzed its association with prognosis in patients with EOC. Consistently, EOC patients with high TIM-3 expression levels in tumor cells had shorter PFS and OS than those with low TIM-3 expression levels. Several studies have elucidated the possible mechanism by which high expression levels of TIM-3 in tumor cells promote proliferation, invasion and metastasis. Xiao et al reported that high expression levels of TIM-3 promote the invasion and metastasis of nasopharyngeal carcinoma cells through activation of the SMAD7/SMAD2/SNAIL1 signaling axis, which mediates epithelial-mesenchymal transformation.^[11]^ Zhang et al demonstrated that high expression levels of TIM-3 in hepatocellular carcinoma cells promote tumor progression through the NF-κB/IL-6/STAT3 axis.^[13]^ Therefore, it is reasonable to speculate that the high expression levels of TIM-3 may affect the survival of EOC patients by promoting the proliferation, migration and invasion of tumor cells. These results suggest that TIM-3 may be a new target for antitumor therapy of ovarian cancer.

Studies have shown that the tumor microenvironment may play an important role in the development of tumors.^[26]^ The tumor microenvironment is composed not only of tumor cells but also stromal cells, including tumor-infiltrating lymphocytes, which may be affected by immune checkpoint molecules, such as PD-1 and TIM-3. Previous studies have shown that TIM-3 mediates Th1 cell apoptosis and inhibits the proliferation of tumor-infiltrating T lymphocytes and the secretion of IL-2, TNF-α, and INF-γ by binding to ligands such as galectin-9, which causes tumor-infiltrating T lymphocytes to exhibit a severely depleted phenotype and enhances the immunosuppressive function of Treg cells.^[27]^ TIM-3 is often coexpressed with PD-1 and some other inhibitory surface markers in exhausted T cells, which is related to the exhaustion of T cells during tumor progression.^[28]^ Several studies have confirmed that high expression levels of TIM-3 in tumor-infiltrating lymphocytes are related to poor prognosis in renal cell carcinoma,^[29]^ gastric cancer,^[30]^ and esophageal squamous cell carcinoma.^[31]^ In ovarian cancer, several clinically relevant trials have reported that high TIM-3 expression levels are associated with high tumor grade and poor prognosis.^[15,16]^ Our study showed that patients with high expression levels of TIM-3 in the tumor microenvironment had poorer PFS and OS than patients with low expression levels. The possible molecular mechanism by which TIM-3 affects the prognosis of tumor patients has also been studied. For instance, Tan et al demonstrated that upregulation of TIM-3 in hepatocellular carcinoma can inhibit the cytotoxicity of tumor-infiltrating NK cells by activating the PI3K/Akt/mTOR signaling pathway and further confirmed in animal experiments that TIM-3 deletion or blocking can enhance the antitumor immunity of NK cells.^[32]^ Another in vitro study showed that TIM-3 expression on differentiated Th1 cells has a repressive role in Th1 immune responses by upregulating galectin-9 when the Th1 immune response is an adaptive immunity response.^[33]^ Currently, several anti-TIM-3 mAbs have been evaluated in clinical trials as monotherapies or in combination with PD-1/PD-L1 mAbs.^[34]^ Hence, TIM-3 is a potential biological target in the immunotherapy of EOC, especially in situations where existing immune checkpoint inhibitors are ineffective.

This study has several limitations. First, although we found that high expression of TIM-3 is associated with poor prognosis in EOC patients, we were unable to reveal the underlying molecular mechanism. Future studies should focus on these aspects. Second, this study is a single-center retrospective study with a small sample size. Expanding the sample size and including patient populations from other centers will enhance the statistical power and the credibility of the conclusions.

## 5. Conclusion

Taken together, our findings revealed that TIM-3 may serve as a potential biomarker for predicting the prognosis of patients with EOC. Moreover, the expression of TIM-3 in the tumor stroma is also of significance and may be related to tumor immune escape in ovarian cancer, although the specific mechanism remains to be further investigated.

## Acknowledgments

The authors acknowledge the doctors at the Department of Obstetrics and Gynaecology, Hebei Medical University, Fourth Hospital, for their assistance in recruiting study subjects.

## Author contributions

**Conceptualization:** Shan Kang.

**Data curation:** Jianlei Wu, Yaru Zhang, Haibo Zhang, Yan Li, Zhihui Jie.

**Formal analysis:** Jianlei Wu, Yan Li, Shan Kang.

**Funding acquisition:** Jianlei Wu.

**Investigation:** Yan Li, Shan Kang.

**Methodology:** Jianlei Wu, Yaru Zhang, Haibo Zhang, Zhihui Jie, Shan Kang.

**Project administration:** Jianlei Wu, Haibo Zhang, Yan Li, Zhihui Jie, Shan Kang.

**Resources:** Jianlei Wu.

**Supervision:** Yan Li, Zhihui Jie, Shan Kang.

**Writing – original draft:** Jianlei Wu, Yaru Zhang, Haibo Zhang, Zhihui Jie, Shan Kang.

**Writing – review & editing:** Jianlei Wu, Yaru Zhang, Yan Li, Zhihui Jie, Shan Kang.
